# *In Silico* Evolution of Gene Cooption in Pattern-Forming Gene Networks

**DOI:** 10.1100/2012/560101

**Published:** 2012-12-25

**Authors:** Alexander V. Spirov, Marat A. Sabirov, David M. Holloway

**Affiliations:** ^1^Computer Science and CEWIT, SUNY Stony Brook, 1500 Stony Brook Road, Stony Brook, NY 11794, USA; ^2^Laboratory of Evolutionary Modeling, The Sechenov Institute of Evolutionary Physiology and Biochemistry, Thorez Prospect 44, Saint Petersburg 2194223, Russia; ^3^Mathematics Department, British Columbia Institute of Technology, 3700 Willingdon Avenue, Burnaby, BC, Canada V5G 3H2

## Abstract

Gene recruitment or cooption occurs when a gene, which may be part of an existing gene regulatory network (GRN), comes under the control of a new regulatory system. Such re-arrangement of pre-existing networks is likely more common for increasing genomic complexity than the creation of new genes. Using evolutionary computations (EC), we investigate how cooption affects the evolvability, outgrowth and robustness of GRNs. We use a data-driven model of insect segmentation, for the fruit fly Drosophila, and evaluate fitness by robustness to maternal variability—a major constraint in biological development. We compare two mechanisms of gene cooption: a simpler one with gene Introduction and Withdrawal operators; and one in which GRN elements can be altered by transposon infection. Starting from a minimal 2-gene network, insufficient for fitting the Drosophila gene expression patterns, we find a general trend of coopting available genes into the GRN, in order to better fit the data. With the transposon mechanism, we find co-evolutionary oscillations between genes and their transposons. These oscillations may offer a new technique in EC for overcoming premature convergence. Finally, we comment on how a differential equations (in contrast to Boolean) approach is necessary for addressing realistic continuous variation in biochemical parameters.

## 1. Introduction

In the pregenomic era, it was a common assumption that complex organisms, with complex body plans and tissue types, had higher genetic complexity than simpler organisms; that unique features corresponded to unique genes. As more and more organisms have had their genomes sequenced, it has become apparent that there are enormous genetic similarities between organisms as diverse as vertebrates, corals and mollusks (see review in [1]). Even in a gene-counting sense, there is little correlation with organismal complexity [2], humans have somewhat more genes than fruit flies or nematodes, but less than pufferfish, cress, or rice [3–5]. If it is not novel genes, what, then, is the source of organismal diversity? It has become increasingly apparent that evolution acts chiefly on gene regulation, on the mechanisms by which a particular gene is expressed or repressed. In the genome, only a small percentage of DNA is involved in coding proteins. It has been realized that the far greater proportion of non-protein-coding DNA (formerly called “junk DNA”) is of critical importance in gene regulation, containing, for example, binding sites for enzymes which activate or inhibit expression of particular genes [6].

The diverse organismal forms we see are generated during the process of development, proceeding from sexual or asexual reproduction to the adult form. Development depends critically on the regulated expression of genes at the correct times and places in order to create an organism's anatomy and physiology. While DNA is commonly referred to as the unique “blueprint” of an organism, current understanding suggests that DNA is far from a catalogue associating specific genes for specific tissue types; rather the genome (especially in eukaryotes) tends to code for relatively few, multifunctional, proteins (~20,000 in humans), along with the markers which enable the genes for these proteins to be regulated in very complex ways. It is this regulation that enables genes to be expressed at the correct times and places. (See special feature on “Describing biology's dark matter” in the September 6, 2012, issue of *Nature*).

In evolutionary terms, this means that a picture is emerging that species (or novel methods for specifying tissues and developmental sequences) do not generally arise due to generation of novel genes. Rather, evolution tends to act on the regulatory sequences of the DNA. Insertion of new regulatory sequences can transfer transcriptional control of a pre-existing gene to other members of the genome [8, 9], and lead to novel patterns of gene expression [10–13]. Existing genes can become new regulators for other pre-existing genes. In developmental pathways, in which networks of genes interact to form particular tissues, cooption (also known as recruitment) of genes from other networks can result in novel dependencies between tissue types, or in new properties of a particular tissue [4, 5].

There are numerous documented cases now of the cooption of genes from one developmental stage to another. For instance, in fruit flies it has been shown how regulatory binding sites in the *yellow* gene were added evolutionarily to control pigmentation patterns in the wing [15]; in sea urchins cooption and optimization of a sequence adjacent to the *spec2a* gene have been elucidated [16]; in brain evolution, the genes involved in vertebrate neural crest cell migration and the midbrain/hindbrain boundary were present in the ancestral chordate—they were coopted into these new roles with the evolution of vertebrates [17]. See also [18, 19]. Indeed, it is commonly thought that early in metazoan evolution, gene networks specifying developmental events may have consisted of no more than two or three interacting genes. Over time, these were augmented by incorporating new genes and integrating originally distinct pathways [8]. In the not so distant past, evolutionary-development research focused on finding phylum-specific genes for phylum-specific features; this has more recently been challenged by evidence that the evolution of body plans proceeds by the changes in gene regulatory circuitries more than by gain or loss of genes [20–22]. Such considerations have led to the view that biological “evolution cannot be fully understood without understanding the evolution of developmental programmes” [23], and such concepts as *developmental reprogramming *[8, 24–26] have been developed to describe the processes lying between mutation and selection at the organismal level (i.e., from an altered gene product (protein) to a new phenotype). Reprogramming should be considered as an evolutionary mechanism because some ontogenetic changes may be promoted by existing developmental mechanisms while others are prevented [23, 27, 28]. It is likely that developmental constraints are powerful factors in the direction of evolutionary change [1, 23, 27, 28].

When considering the evolution of developmental programs, one needs to ask what the constraints are; that is, if change occurs in a gene regulatory network, by what measures is the new program tested with respect to its fitness? Development needs to be robust to numerous factors, such as environmental temperature, egg size, dosage of maternal regulatory molecules, intrinsic noise in gene expression, and variability in cell geometry and cell order. Developmental networks must optimize fitness to all of these challenges (and more). Strong genetic or environmental perturbations can induce increased phenotypic variance [29–32]. Waddington introduced the concept of canalization to describe how wild type (normal) development buffers against such perturbations, such that the developmental program tends very strongly to achieve a well-defined end result, despite perturbations which may cause some diversity in the paths that reach that end point. Quantitative experiments are beginning to demonstrate canalization in developmental sequences (diverse trajectories to a precise end point, e.g., [33, 34]). However, there are still large unknowns regarding what specifically makes given networks robust, and exactly how such networks have evolved. Since developmental events generally involve very complex gene network dynamics, frequently in concert with cell-cell communication and tissue mechanics, computational modeling is a key tool in understanding not only how such processes operate (for instance, to generate spatial domains of gene expression), but what in their dynamics confers robustness to diverse perturbations. In addition to studying gene network function, computation can allow us to test how networks arise through evolution. In concert with experimental data, this can address specific questions regarding how evolution operates on gene regulation, and how network evolution contributes to developmental robustness.

Early segmentation of the invertebrate body plan has long been very popular for studying the specifics of both developmental mechanisms and evolution [35, 36]. As reviewed in [36] it appears “that throughout evolution there was a parallel cooption of gene regulatory networks that had conserved ancestral roles in determining body axes and in elongating the anterior-posterior (AP) axis. Inherent properties in some of these networks made them easily recruitable for generating repeated patterns and for determining segmental boundaries. Phyla where this process happened (arthropods, annelids, and chordates) are among the most successful in the animal kingdom, as the modular nature of the segmental body organization allowed them to diverge and radiate into a bewildering array of variations on a common theme.”

For instance, the Notch and *wnt* pathways have ancient roles in axis elongation. Discovered and most intensively studied in the fruit fly *Drosophila melanogaster*, these genes began forming periodic spatial patterns somewhere along the lineage to arthropods. These periodic patterns have now come to underlie segmentation in numerous phyla. Some genes such as *engrailed* (*en*) had a primitive role in neural patterning (which is also segmental) and appear to have been coopted to body axis segmentation (a classic example of boundary formation in *Drosophila* involves *en* and the *wnt* pathway). In fact, a number of the segmentation genes appear in both neurogenesis and segmentation [37, 38], including the *hunchback *(*hb*) and *Kruppel* (*Kr*) gap genes covered in more detail below [39–41]—it may be the nervous system that provides a large reservoir of useful components that have already been tested in gene networks. *Even-skipped *(*eve*), a gene upstream of *en* (in *Drosophila* development) was involved in axial elongation and became coopted into segmentation, see [36]. *Caudal* (*cad*) is involved in axis elongation in many invertebrates, but was shown to have evolved a role in generating segmental periodicity in the centipede *Strigamia maritima* [42, 43]. This role for *cad* is clearly derived, but its recruitment to this role would have been facilitated by it already being expressed in the segmenting tissue at the correct time during development.

In insects, two distinct modes of segmenting the body have evolved. In primitive insects, such as the grasshopper, the short-germ band mode lays out body segments sequentially. Many more highly derived insects, such as flies, use the long germ band mode to establish all body segments simultaneously. This simultaneous mechanism must act quickly during development; it has been proposed that it evolved by cooption of new genes to the short-germ band mechanism, in order to maintain accurate regulation of patterned gene transcription over the whole embryo in a condensed time frame [8]. This complex task appeared to be solved by evolution in a short geological span at the sacrifice of, as a minimum, doubling the number of genes in the segmentation network. As doubling occurs, genes from other gene ensembles are often recruited into the network.

As well as the wealth of comparative information on the evolution of segmentation, the molecular biology and genetics of development are extremely well characterized in insects, particularly *Drosophila*. This has allowed for the development of very detailed quantitative models of the developmental mechanisms involved in segmentation. These models can be used to study both the functioning of the segmentation network, and how it evolved (in concert with comparative data between species); see [33, 34, 39, 44–48].

Segmentation in *Drosophila* is temporally hierarchical. In this paper, we focus on the earliest stages, the maternal, and gap gene patterning. Maternal factors (mRNAs and proteins) form monotonic concentration gradients along the AP axis. These products are transcriptional regulators, and their first targets are the zygotic gap genes, which form broad expression domains. We work with a gene circuit model of 4 trunk gap genes (adapted from [33, 34])—(*hb*; *Kr*;* giant*, *gt*; *knirps*, *kni*) under the control of the maternal Bicoid (Bcd) gradient. See the HOX pro Web resource [49, 50] for a catalogue of the known regulatory elements for the trunk gap gene ensemble. We model the central part of embryo only, from 34% to 82% egg length (%EL, head to tail), where contributions from terminal regulatory networks can reasonably be neglected (see [51, 52]). Models of this small network have been fit in detail to *Drosophila* expression data and serve as a starting point for exploring how this network may have evolved and what this can say in general about evolutionary mechanisms.

Our chief focus in this project is to study how gene recruitment affects network structure and dynamics, and what this implies for developmental robustness. The 4-gap gene model provides a small very well characterized network for investigating this. In this study, we specifically focus on robustness to maternal perturbations. That is, we test to what degree the gap gene expression patterns are robust to variability in the maternal Bcd gradient. In earlier work, we optimized gap gene models for robustness to naturally occurring levels of Bcd variability [33, 34]. In the current project, we use these models as a starting point for evolutionary computations to study gene recruitment. We can ask if the current *Drosophila* 4-gap network is optimal for this type of robustness, or whether recruitment of additional genes could increase robustness. We can also study how the 4-gene network may have evolved; for example, by starting with 2-gene models, we can study how these might have recruited genes into the current network. With computation, we can study many aspects of such a process. Does recruitment increase robustness? If so, what types of genes are recruited, that is, in what ways do they connect into the original network; what types of expression patterns might they have (do they recapitulate known patterns or form novel ones)? How fast does recruitment occur? (I.e., what is the relation between recruitment and evolvability?) However, at the same time that we want to address such questions regarding the evolution of development, many of the basics of how recruitment occurs are poorly understood. For instance, some studies assume that recruitment occurs occasionally, by chance, and is then subject to evolution; others believe that there are special evolutionary mechanisms for recruitment. By trying different recruitment scenarios, a computational approach can address such questions as do different means of recruitment lead to more robust networks, or larger networks, or faster evolving networks?

A large diversity of methods have been developed in recent years to model evolution. These range from techniques inspired by biological evolution but used for diverse optimization problems (e.g., in engineering), such as Genetic Algorithms (GA) and evolutionary computations (EC) generally [53], to techniques which have been developed specifically for studying the mechanisms of biological evolution (*in silico* evolution). A new research program in evolutionary systems biology is beginning to arise through the fusion of systems biology, network theory, and evolutionary theory. Within this, a number of groups have developed computational approaches for the evolution of gene regulatory networks (GRNs), evolving populations of individuals represented by dynamically modeled transcriptional regulatory networks. Some examples include work on the evolution of robustness and evolvability [7, 54–63], work on the mechanisms of genetic assimilation [64]; study of the role of network topology [65], and computational investigations into gene duplication and subfunctionalization [66]. Wagner's model in particular has helped elucidate why mutants often show a release of genetic variation that is cryptic in the wild type (Waddington's canalization), and how adaptive evolution of robustness occurs in genetic networks of a given topology [7, 54, 56, 60, 63]. Variants of this model have proven useful for studying the evolution of modularity in gene circuits [67] and the evolution of new gene activity patterns [61, 68, 69]. Also see [70–73].

In this work, we develop EC techniques for studying mechanisms of gene cooption. Following earlier work [71], one approach is to add Gene Introduction and Gene Withdrawal operators to a standard GA algorithm (running repeated cycles of mutation, selection, and reproduction). These give a probability to adding (or subtracting) a gene to a given network (a random cooption event), along with its attendant connectivities to the other network genes. This approach is at the network level (i.e., with genes as the fundamental units, or network nodes). We have also developed approaches at the next level of detail, studying the evolution of regulation at the DNA level. At this level, we can begin to characterize the dynamics of particular mechanisms of regulatory evolution. For instance, we have improved GA optimization speeds by developing crossover operators inspired by the mechanisms of retroviral recombination [75, 76]. Here, we describe a technique to model genetic change due to transposons (also see [77–83]).

A great deal of the so-called junk DNA is comprised of intermediate repeats of DNA elements that are able to move (or transpose) throughout the genome. Transposons are ubiquitous and may comprise up to 45% of an organism's genome. Transposons jump between different parts of a genome to propagate themselves, and these events are usually to the detriment of their host [6]. Many transposons have a unique DNA site that acts as a forwarding address and directs the transposon to a complementary DNA site in its host genome [6]. It has been estimated that 80% of spontaneous mutations are caused by transposons [6]. Changes include the creation of novel genes, the alteration of gene expression in development, and the induction of major genomic rearrangements [84–86]. Transposable elements are ubiquitous among contemporary organisms and have probably existed since the dawn of life. Transposons can be viewed as parasites that have coevolved with their hosts, over time introducing useful variations into host genomes. Transposons are natural tools for genetic engineering [87]. Since transposons are likely to be active players in the rewiring of preestablished regulatory networks, we are interested in characterizing the dynamics of transposon-induced evolution of GRNs. We can imagine that transposons may be more effective agents of change than local random mutations, since transposons can deliver large sequences of DNA (whole genes or regulatory regions). By better understanding a major mechanism of biological evolution we may be better able to use it for optimization problems or for directed evolution experiments (e.g., see [88, 89]). Transposons also allow us to study the “arms-race” aspect of the coevolution of the host GRN and the “parasitic” transposons. 

Our approach uses continuous PDE models of the evolving gap GRN (i.e., gene product concentrations and regulator strengths are represented as real numbers). Prior publications in GRN evolution have tended to use discrete models (e.g., Boolean approaches in which a gene is “on” or “off”). We are able to move beyond the knock-out mutations and/or abstract environmental stochasticity used in discrete models and address continuous variation in gene products, such as continuous variation in the maternal gradients. Through this, we can ask questions such as the following: how far can any particular component in the wild type be varied before an alternate phenotype is accessed; and with continuous variation, are the observed transitions between phenotypes continuous or discrete?

**Figure 1:**
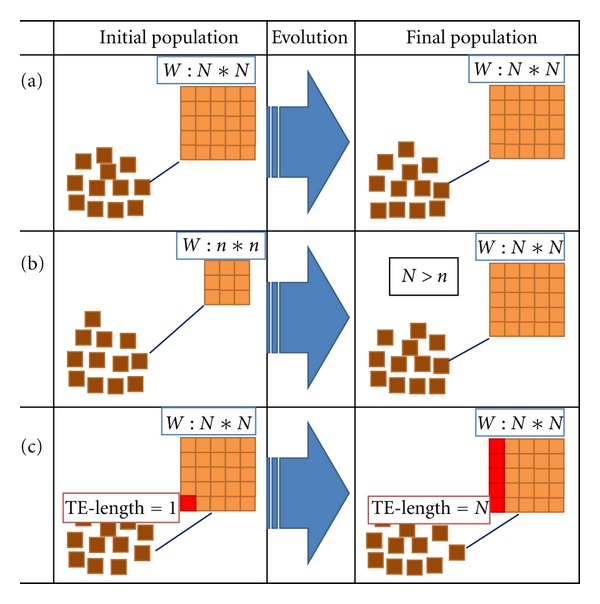
Three ways to introduce gene recruits for cooption in *W* matrix-based models (gene-gene interaction, see Figure 2 below) of GRN evolution *in silico*. (a) Static determination of recruits. Initial population has fixed numbers of obligatory (2) and additional (recruited) genes. Only the obligatory genes are used to fit the data. The number of the recruits does not change in a given GRN or its descendants during evolution. The *W* matrix dimension here is *N* × *N*. (b) Dynamic addition of recruits. A Gene Introduction operator adds new genes to the GRNs during the evolutionary search. Gene Introduction can be complemented by a Gene Withdrawal operator. (c) Dynamic evolutionary forcing by transposons. A set of transposon and transposition operators forces evolutionary search via a restriction of evolutionary space (e.g., by zeroing the elements of the *W* ^*a*0^ column of the *W* matrix, Figure 2). With evolutionary time, transposons (transposable elements, TE) form one-dimensional clusters of length *N* (TE-length = *N*).

**Figure 2:**
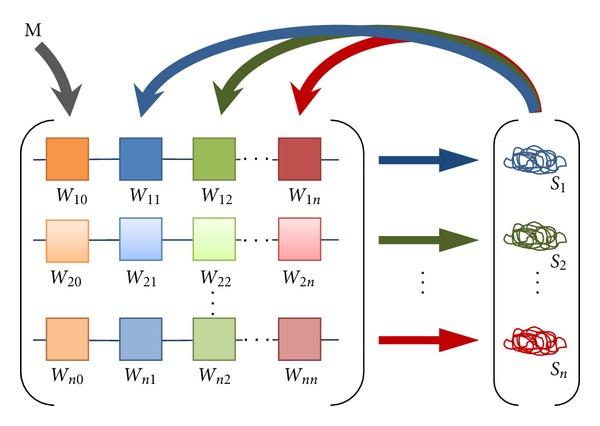
The gene-gene interaction matrix, a core element of the coarse-grained modeling of gene regulatory networks. Each gene (horizontal arrow) is regulated by the products of other genes via upstream enhancer elements (boxes). The strength and direction of regulation (depicted as differently colored saturation levels) are a function of both the regulatory element and the abundance of its corresponding gene product. The left-most column *W* ^*i*0^ corresponds to the regulatory elements for the action of the morphogen, M = Bicoid (external factor for the GRN). The genotype is represented as the matrix, *W*, of the regulatory interactions, and the phenotype is the vector, Ŝ, of the gene product levels at equilibrium. Modified after [7].

In this publication we consider four scenarios for gene cooption (Figure 1). Two of these are at the network level: *static determination of recruits*, in which an evolutionary search has a fixed number of potential genes to recruit from the population (i.e., all individuals have the same number of genes—two obligatory initial genes and N-2 recruits—and this stays constant during the evolutionary search); *dynamic addition of recruits*, in which the Gene Introduction and Withdrawal operators are used and produce gradual changes in population-average number of additional genes in the evolved population. The static determination provides a baseline against which to understand the effect of dynamic recruitment. At the more detailed level of DNA regulation, we consider two mechanisms for cooption, via transposons and transposition operators. These are as follows: *static transposon tests*, in which all individuals in a population keep the same transposon at the same location (in terms of the discussion below, Figure 2, this is the *W* ^*a*0^ column of the *W* matrix) and the transposon permanently forces evolution (by keeping the *W* ^*a*0^ elements predetermined); *dynamic evolutionary forcing by transposons*, in which transposon and transposition operators gradually enlarge the population-average length of transposons in the evolved population. In this case the evolutionary pressure by transposons rises with evolutionary time.

We test these four mechanisms for their ability to coopt genes into existing developmental GRNs to alter function. We use the specific case of maternal gradient reading in the *Drosophila* segmentation network, for which we can calibrate network dynamics against quantitative data. Within this system, fit GRNs are those which create precisely positioned gap gene domains despite variability in the maternal gradient. Robustness to maternal factor variability is a key feature of developing systems and has spurred a great deal of interest in the biology community with respect to how embryos might achieve this [90–95]. Through EC, we are aiming to characterize what some of the key factors might have been in evolving GRNs with such robustness.

In simulations we can alter and evolve thousands of GRNs, a subset of which may be robust to maternal variability. Analysis of these solutions allows us to compare the efficiency of different cooption mechanisms (e.g., random mutation versus transposons), and whether particular mechanisms may favor particular types of recruits, in terms of, say, their expression patterns or network connectivity and how they affect network behavior. Discussion in this area has predicted that growth of GRNs via cooption should cause both structural (genes duplicating existing ones) and functional (development of compensatory pathways) redundancy [96]; this has been observed in a number of organisms [96]. Such redundancy is likely to affect characteristics such as evolvability (ability of the network to change) or robustness to perturbations and variability during development. Our simulations offer a direct way of characterizing such interrelations. 

## 2. Methods and Approaches

The mutual inhibition of the 4 trunk gap genes (*hb*, *Kr*, *kni*, *gt*) plays a major role in establishing their expression domains. Models with all 4 genes capture most of the features of gap expression domains. However, the dynamics have also been broken down and studied in terms of mutually inhibitory pairs, such as Kr-gt and kni-hb (e.g., [39–41, 97]). While some subnetworks of the 4 genes can recapitulate major features of the trunk pattern—for instance, addition of Kr to a Bcd-hb subnetwork confers robustness of hb pattern to Bcd variability [71]—other combinations will not. For instance, a Bcd-Kr-kni subnetwork is not sufficient to form gap patterns. We will use this feature to study the role of cooption in the gap network. By making only Kr and kni obligatory in the starting networks, we can create a tendency for the network to coopt additional genes in order to meet the criteria of forming normal gap patterns (as evaluated by fitting model output to experimental Kr and kni patterns). We can think of the obligatory Kr and kni genes as an “ancestral” network, which evolution needs to enlarge in order to solve the problem of simultaneous gap patterning. 

### 2.1. Regulation Matrix-Based Modeling of the GRN

The network is represented at the coarse-grained “gene circuit” level [98]; the dynamics of each gene product (protein) *a* in each nucleus *i* (1 nucleus ~1%EL in distance) is given by a system of *number of proteins* times *number of nuclei* ODEs (Ordinary Differential Equations) of the form

(1) $$\begin{array}{c} \frac{\partial \nu_{i}^{a}}{\partial t}=R_{a}g\left(u^{a}\right)+D_{a}\Delta \nu_{i}^{a}-\lambda_{a}\nu_{i}^{a}. \end{array}$$

The main terms on the right hand side of (1) represent protein synthesis (*R*_*a*_), diffusion (*D*_*a*_, Δ) and decay (*λ*_*a*_). *g*(*u* ^*a*^) is a sigmoid regulation-expression function. For values *u* ^*a*^ below −1.5*g*(*u* ^*a*^) rapidly approaches zero and above 1.5 approaches unity. *u* ^*a*^ is given by *u* ^*a*^ = ∑_*b*_ *W* ^*ab*^ *v*_*i*_^*b*^ + *h* ^*a*^. The genetic interconnectivity matrix, *W* ^*ab*^, is the key component describing the gene-gene connections and their strengths (Figure 2). The *W* ^*ab*^ elements represent the activation of gene *a* by the product of gene *b* (with concentration *v*_*i*_^*b*^) if positive, repression if negative, and no interaction if close to zero. *h* ^*a*^ represents regulatory input from ubiquitous factors.

### 2.2. Experimental Data for Fitting

**Figure 3:**
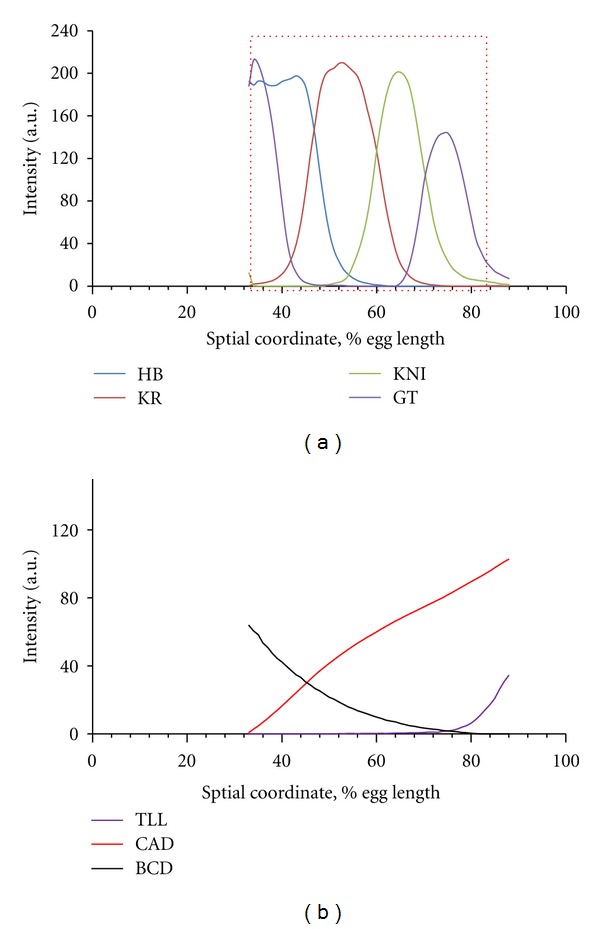
Biological data used to fit ODE model by GA. Integrated gene expression profiles for mid nuclear cleavage cycle 14 A. Vertical axis, relative protein concentration (proportional to intensity); horizontal axis, relative position along the anteroposterior (AP) embryo axis (0% is the anterior pole). Data from the FlyEx database [14]. (a) Protein profiles for four trunk gap genes (giant; gt, hunchback, hb; Kruppel, Kr; & knirps, kni). The red rectangle marks the part of the AP axis modeled in this publication. Models are fit to the Kr and kni data. (b) Profiles of two proteins which are external inputs in our simulations (the primary morphogen Bicoid, Bcd, and the transcription factor Caudal, Cad).

We fit our model results to data from a large-scale project we were engaged in to collect, process, and analyze the expression of the *Drosophila* segmentation genes [91, 94, 99]. This FlyEx dataset is now available publicly [14]. In this paper, we use expression data from mid nuclear cleavage cycle 14 (prior to full cellularization), the developmental stage during which segmentation patterns become mature.  Figure 3 shows an example of this data for the 6 gene products in our model (maternal proteins Bcd and Cad and the 4 gaps). Models (in this publication) are evaluated by the quality of their fit to the Kr, kni data (Figure 3(a)).

Within the framework described in Section 2.1 (1), we model gap gene cross-regulation and their control by up to two nongap transcription factors: the primary morphogen Bicoid (Bcd) (Sections: 3.1–3.5 results); and the transcription factor Caudal (Cad) (Section 3.6: results). 

### 2.3. Evolutionary Computations to Simulate Evolution of GRNs

The set of ODEs (1) representing the gap GRN was solved numerically by Euler's method [100]. A cost function was calculated from the difference of the output model gene product concentrations and the corresponding experimental concentrations:

(2) $$\begin{array}{c} E=\sum_{b}{\left({v_{i}^{a}}_{\text{model}}-{v_{i}^{a}}_{\text{data}}\right)}^{2}. \end{array}$$

Evolutionary computations (EC) were run on the elements of the interaction matrix *W* ^*ab*^ to minimize the cost function *E*. The other model parameters, *m* ^*a*^, *h* ^*a*^, *R*_*a*_, *D* ^*a*^, and *λ*_*a*_, were found in preliminary runs and then used as fixed parameters. EC followed the general scheme of population dynamics (common to both GA [101] and general simulations of biological evolution), with repeated cycles of mutation, selection, and reproduction. Following the standard GA approach, the program generated a population of floating-point chromosomes, one chromosome for each gene *a*. The value of a given floating-point array *a* (chromosome *a*) at index *b* corresponds to a *W* ^*ab*^ value. 

Initial chromosome values were generated at random. The program then calculated the *ν*_*i*_ by (1) and scored each chromosome set (*W*matrix) by the cost function *E* (2). An average score was then calculated for all the chromosome sets run. Chromosome sets with worse-than-average scores were replaced by randomly chosen chromosome sets with better-than-average scores. A portion (40%) of the chromosomes were then selected to reproduce, undergoing the standard operations of mutation and crossover (defined below), changing one or more of the *W* ^*ab*^ values. The complete cycle of ODE solution, scoring, replacement of below-average chromosome sets, and mutation and crossover was repeated until the *E* score converged below a set threshold, typically 50–100 generations. (In case convergence did not occur, all computations were stopped by EvalSum = 1,000,000 evaluations.)

In GA, mutation is a genetic operator used to maintain genetic diversity from one generation of a population of chromosomes to the next, analogous to biological mutation. Point mutation in GA involves a probability that a *W* ^*ab*^ value on a chromosome will be changed from its original state (comparable to changing a nucleotide in biological point mutation). Upon mutation, a *W* element is updated according to [*W* ^*ab*^] = [*W* ^*ab*^] ± ln⁡(Random(Power)), where Power = 1,000,000.

GA crossover is a genetic operator used to vary chromosomes from one generation to the next, by swapping strings of values between chromosomes, analogous to crossover in biological reproduction. We use one-point crossover in this study, in which a point on a parent chromosome is selected, then all data beyond that point is swapped between two parent chromosomes.

The model is implemented in Delphi (Windows) and Free Pascal (Linux) and available from the authors upon request.

#### 2.3.1. Introduction and Withdrawal of New Genes

**Figure 4:**
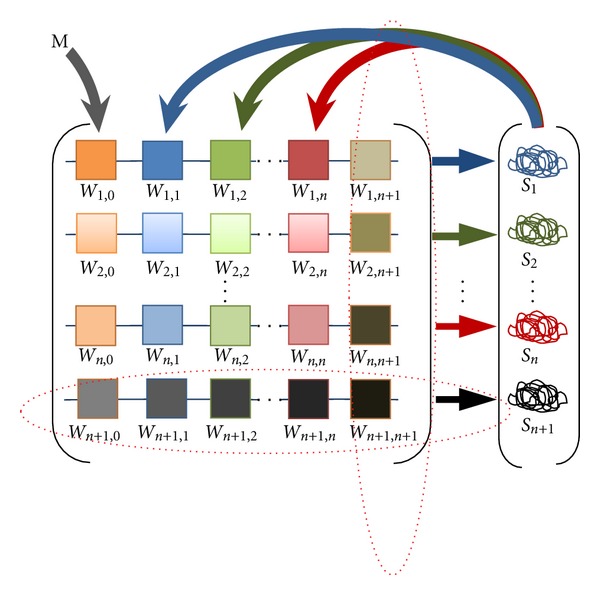
Gene Introduction adds a new (*n* + 1) column (right-most) and row (lowest) to the *W* interaction matrix. Gene Withdrawal eliminates the right-most column and bottom row.

As a first way of modeling dynamic recruitment of genes to the gap network, we introduce new GA operators for Gene Introduction and Gene Withdrawal. Gene Introduction adds a new gene to the network at a rate of 5–10% per generation (depending on the simulation). Specifically, this adds a new row and column to the *W* ^*ab*^ matrix (Figure 4), which can be then be operated on by mutation and crossover. To balance this process and control the number of genes in the network, Gene Withdrawal removes a row and column from the *W* ^*ab*^  matrix (at a rate of 2–10% per generation, depending on the simulation). Gene Withdrawal does not operate if the network is minimal (*N* = 2 genes). Since Gene Introduction simply adds a gene to the network, which then adapts, it does not distinguish between the biological cases of a new gene arising by duplication or of an existing gene being recruited from another network [1, 3]. Two parameters control the Introduction and Withdrawal procedure. The ToRecruitingProc parameter defines what part of population (from 0 to 1) will be subjected to the procedure. Another parameter, WithdrAdd (from 0 to 1) specifies the probability that a given solution (*W* matrix) will be subjected to Gene Withdrawal or Gene Introduction. (If WithdrAdd = 0 then all solutions will go through Gene Introduction only; If WithdrAdd  =  1 then all solutions will go through Gene Withdrawal only.)

#### 2.3.2. Involvement of the Recruited Gene in the Functioning of the GRN

To quantify how much added genes affect network fitness, we used the following procedure: the model solution in a given generation was evaluated according to (2); then, for each additional gene (above the obligatory 2), fit to the data was evaluated with the *W* elements for that gene zeroed out. In cases where this produced a drop in fitness score (2) of more than 10% (a threshold determined in preliminary runs) compared to the full GRN, we kept the added genes as recruits to the GRN. We further filtered the most functionally significant recruits by use of a 33% threshold. Results are presented below for both threshold levels.

#### 2.3.3. Evaluation of GRN Robustness

GRN solutions with *E* scores below threshold represent good fits to the gene expression data (Figure 3(a)). That is, these GRNs solve the problem of forming gap expression domains. In addition to this, though, we want to test the robustness of these gap solutions to maternal variability. To do this, we took each good solution and tested its robustness to Bcd variability. We perturbed Bcd from Figure 3(b) according to

(3) $$\begin{array}{c} \left[bcd\right]=\left[bcd\right]\pm \left[bcd\right]*\text{Random}\;\left(0.2\right)\; \end{array}$$

(i.e., the Bcd profile varied within limits of ±20%). We reran the GRN with the perturbed Bcd values and compared these against the unperturbed result according to

(4) $$\begin{array}{c} E^{\prime }=\sum_{b}{\left({v_{i}^{a}}_{\text{perturbed}}-{v_{i}^{a}}_{\text{unperturb}}\right)}^{2}. \end{array}$$

This measure was calculated for 100 Bcd perturbations for each GRN, and the results averaged for a measure of the GRN's robustness (e.g., Figure 12).

#### 2.3.4. Artificial Transposons for GA

The above Gene Introduction operator does not model the mechanism by which genes are incorporated into the genome. To begin addressing this, we have developed a model for transposon dynamics in our simulations. We define an artificial transposon as a marked block of the host's code. The mark is transmittable from host to host. For this, we use double-string chromosomes, in which the main string (floating point) is used for the host codes, while an additional string (binary) is used for the transposon marks (1 denotes a transposon mark; 0 is unmarked):

(5) the  additional  string:  1 0 0 ⋯ 0the  main  string:  a1 a2 a3 ⋯ an,

where *a*_*i*_ are host code floating-point values (only the *a*_1_ element is transposon marked in this example).

#### 2.3.5. Artificial Transposons as Mutators

**Figure 5:**
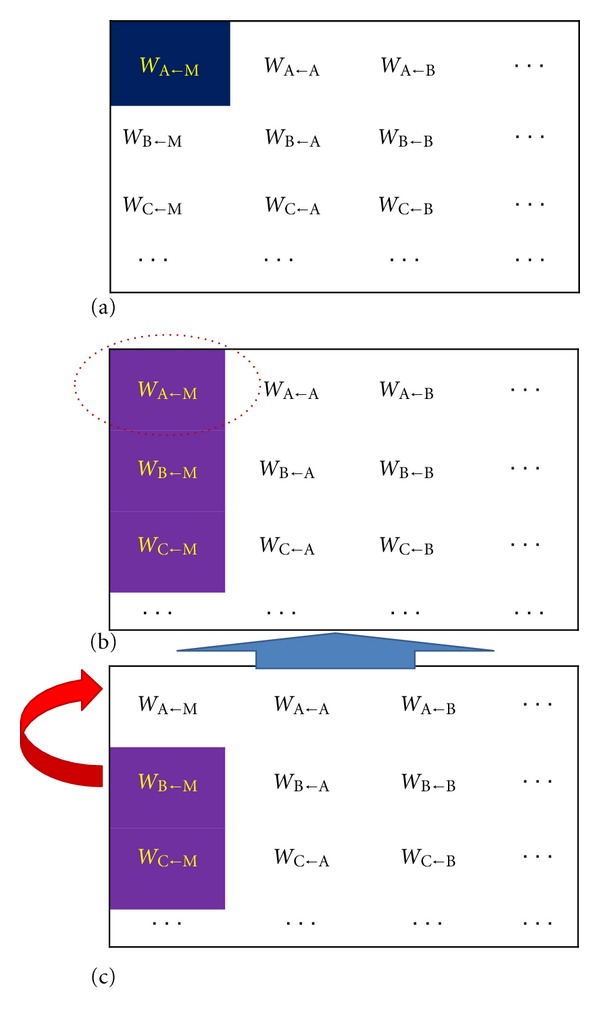
Artificial transposons (a) and their mechanism of spreading (b-c).

 As with biological transposition, an artificial transposon tends to be deleterious to the host. To see how this affects the *W* interaction matrix, consider the following: let the first-row, first-column element of *W*_*A*←*M*_ be infected by a transposon (Figure 5(a), highlighted). The transposon's deleterious action is then implemented by decreasing the value of the infected host element. Specifically, we halve the *W*_*A*←*M*_ value in each generation. This quickly drops the element value to near zero. In this manner, the transposon effectively cuts the *A* ← *M* regulatory connection.

#### 2.3.6. Spread of Artificial Transposons

Transposons tend to form clusters in host chromosomes. We simulated this feature by spreading transposon infection by at most one element per generation. In this operation a transposon can mark the *W* ^*j*←*i*^ element above it as a new transposon. (Figures 5(b) and 5(c)).

#### 2.3.7. Transmission of Transposons

We used fixed transposon coordinates to transmit transposons from host to host. (Whole transposons were never moved along the chromosomes.) The two-place transmission operator was implemented as follows: first, a pair of hosts was chosen at random; then a chromosome from either host was scanned for transposon marks. If a transposon was found, it was replicated in the partner chromosome, regardless of the original string character in the target chromosome. Copying only occurred if the secondary strings had transposon marks.

## 3. Results

In general, we found recruitment of new genes to a preexisting GRN to be typical; we saw this trend in all of the evolutionary computational designs implemented (Figure 1). Recruited genes can be uniform or spatially patterned. These patterns can either recapitulate patterns of existing network genes or introduce patterns novel to the model network (but like patterns seen in the full biological gap network).

### 3.1. Parameter Optimization for the Evolutionary Computations

**Figure 6:**
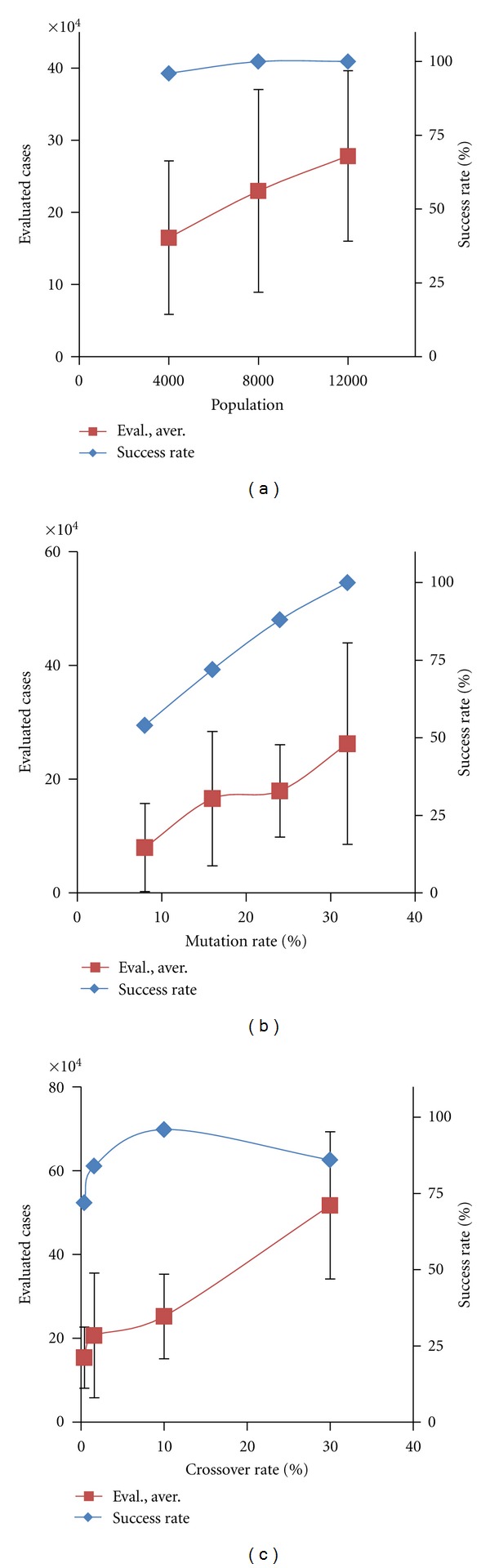
Dependence of evolutionary search on several key computational parameters. (a) Dependence on population volume (Popul = 4,000; 8,000; 12,000). (Other parameters were as follows: Reprod = 40%; Mut = 38%; EvalSum = 1,000,000; Cross = 2%; Power = 1,000,000.) Success rate is the percentage of runs achieving the desired fitness level. (b) The dependence on mutation rate (Mut = 40%, 32%, 24%, 16%).  (c)  The  dependence  on  crossover  rate (Cross = 0.5%, 2%, 10%, 30%). (50 runs for each point).

In order to compare x we established standardized settings for a number of the computational parameters. We have found that the most important parameters for fast evolutionary searches are population volume, Popul; reproduction rate (quota of population to be replaced by offspring in each generation), Reprod and the mutation and crossover rates (Mut and Cross, resp.). In preliminary tests, we found a reasonable value of Reprod to be 0.4 (i.e., in each generation 40% of the population is subjected to reproduction by a truncation strategy). The most effective population volume depends on the number of genes recruited: for 4 genes, best results were at Popul = 8000; for the 8 genes, best results were for Popul = 12,000 − 16,000 (Figure 6(a)). For mutation, we tested Mut values of 40%, 32%, 24%, and 16% (with Popul = 8,000, Reprod = 40%; EvalSum= 1,000,000; Power = 1,000,000, Cross = 2%; and 4 recruited genes); see Figure 6(b). Best results were achieved for Mut = 40% and 32%. For crossover rate, we tested Cross = 2%, 4%, 10%, and 20% (with Popul = 8,000; Reprod = 0.4; Mut = 20%; EvalSum = 1,000,000; Power = 1,000,000; and 4 recruited genes); see Figure 6(c).

### 3.2. Static Recruitment: GRN Evolutionary Complexification without External Forces

We ran gene cooption computations with 3 different mechanisms (see Figure 1 and Sections 3.3 and 3.4). All scenarios start from the obligatory Kr-kni system and add genes to improve the fit of the computations to the experimental expression data for Kr and kni. As a control, our simplest scenario is to have all additional (nonobligatory) genes already in the *W* matrix. Genes are neither introduced nor withdrawn from the matrix, but the *W* ^*ab*^ elements of the nonobligatory genes adapt over the course of the computations in order to improve the fit to the data. We expected the additional genes to become increasingly incorporated (necessary) in the pattern-forming network, since Kr-kni alone is insufficient for proper patterning. We considered cases from two to eight additional genes (*W* matrices of dimension 4 to 10). These computations allowed us to estimate some key evolutionary parameters for comparison with the directed (forced) evolution cases below (Sections 3.3 and 3.4) such as the following: (a) how fast can evolution find the desired Kr and kni patterns (evolvability), (b) how many genes on average are recruited to the networks (and what proportion of these recruits are highly involved in the network), and (c) how robust are the networks to Bcd variability.

**Figure 7:**
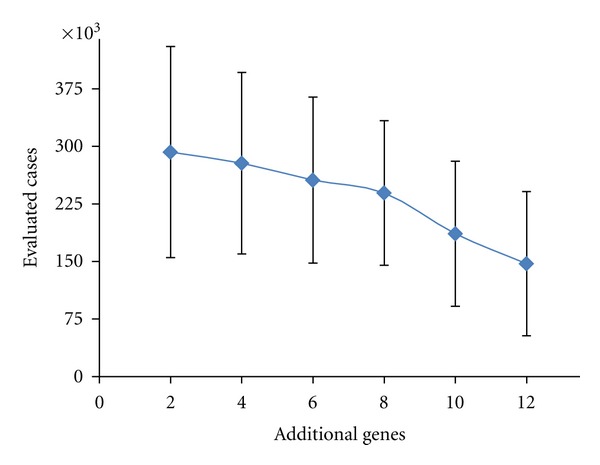
The dependence of evolvability (speed of evolutionary search) on the number of genes recruited to the initial population (the two obligatory genes). Results are shown for 2, 4, 6, 8, 10, or 12 additional genes. Average evolvability (lower values indicate faster evolution) is plotted against the number of recruits in the *W* matrix.

The results on evolvability are shown in Figure 7 for *W* matrices of dimension of 4, 6, 8, 10, 12, and 14. (We show a minimum of two additional genes, since preliminary runs indicated that a single additional gene was not sufficient to improve the Kr-kni fit.) First, we see that all solutions with good fitness scores tend to recruit all available additional genes, and that the functional involvement of the recruits in the GRN is very high (even in this simple static scenario), with a negligible difference between the 10% and 33% selection thresholds. Second, evolvability slightly but steadily improves (networks become more evolvable) with the dimension of *W* (the number of available recruits). 

### 3.3. Dynamic Addition of Genes: Introduction and Withdrawal Operators

Building on our previous work [71], we have found that addition of new genes during an evolutionary search (enlargement of the *W* matrix, followed by adaptation of the *W* ^*ab*^ elements) is an effective way to enlarge networks with the desired functionality. With static recruitment (Section 3.2), we found that GRNs tend to incorporate all available genes in a functional manner. With dynamic control, we similarly find that networks tend to incorporate genes and enlarge. A simple explanation may be that new recruits create an implicit pressure on the network new genes arrive with zero *W* ^*ab*^ values, and it is far more likely for these *W* ^*ab*^ values to evolve away from zero, incorporating the new gene into the functioning of the network, than to maintain the *W* ^*ab*^ at their initial zero values. Functional incorporation of genes into the network (in the dynamic scenario) should depend on the rate of the Introduction operator. To test this, we can tune the rate of the Withdrawal operator to control the net Introduction rate. Recruitment should be favored when the Withdrawal/Introduction ratio is low (Introduction probability ≫ Withdrawal probability; the implicit pressure is high) and reduced when the ratio is large.

**Figure 8:**
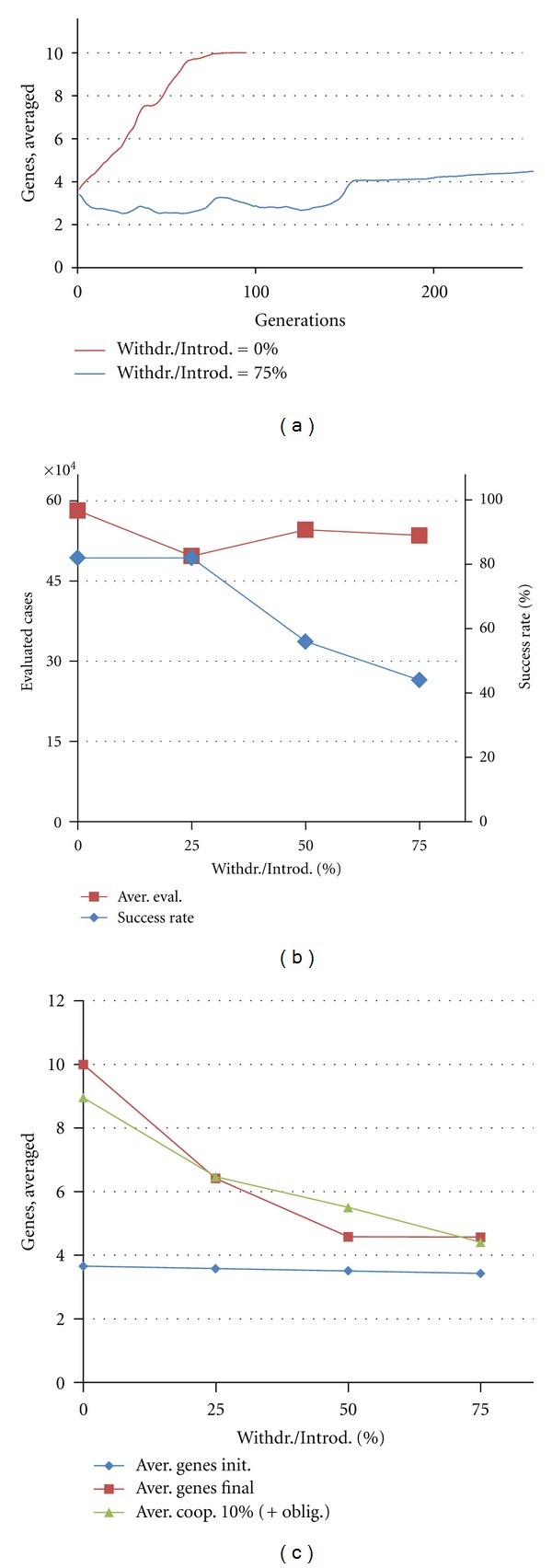
Effects of the Gene Withdrawal-to-Introduction ratio. Results are averaged over 50 runs for each Withdrawal/Introduction value. (a) Recruitment—growth of the number of genes recruited (population average) during computational evolution without Withdrawal (0.00) and with high relative Withdrawal rate (0.75). (b) Evolvability—Evaluated cases and success rate against the Withdrawal/Introduction rate. (c) Growth of networks during evolutionary search at different Withdrawal/Introduction rates: population average of genes in initial GRNs, population average of genes in final GRNs, and average functionally coopted recruits (10% threshold). Nearly all recruits are functional. Other parameters are as follows: Popul = 8000; Reprod = 40%; potential recruits = 8; Mut = 32%; Cross = 8%; EvalSum = 1000000.

The tendency towards recruitment can still occur for Withdrawal/Introduction ratios more than 50% (more Withdrawal cases than Introduction cases), due to the effect of random mutations (which can add genes). With the mutation rates used in our simulations, recruitment begins to shut off for ratios less than 1/3 (Introduction probability = 25%; Withdrawal probability = 75%). By tuning these parameters, we can characterize their effect on network outgrowth (addition of genes) and evolvability (Figure 8).

We see that higher relative Withdrawal rates slow the rate of network outgrowth (Figure 8(a)), and also decrease the success rate (Figure 8(b)). The decrease in network size due to the Withdrawal rate does not appear to be affected by the cooption threshold (Figure 8(c)).

### 3.4. Forced Evolution by Artificial Transposons

Here we begin to model a biological mechanism for the introduction of genes to the network, by the action of transposons. As introduced in Section 2.3.5, our mechanism for artificial transposition can effectively shut down the input into the GRN from a particular regulator. As illustrated in Figure 5, for example, transposon infection can cut out influence of the maternal regulator (Bcd). Transposon infection, computationally, is a means of restricting the search space of the evolutionary problem.

#### 3.4.1. Static Transposons

As a control for studying dynamic transposon infection, we have run a series of computations with statically “knocked out” regulators, that is, *W* ^*ab*^ in which an entire column is zeroed or held constant. We have run a series of computations in which the first column (for maternal regulation) is held constant while the rest of the matrix is free to evolve.  Table 1 summarizes these, giving the constant *W* ^*a*0^ values run for each matrix dimension (number of genes) run.

**Figure 9:**
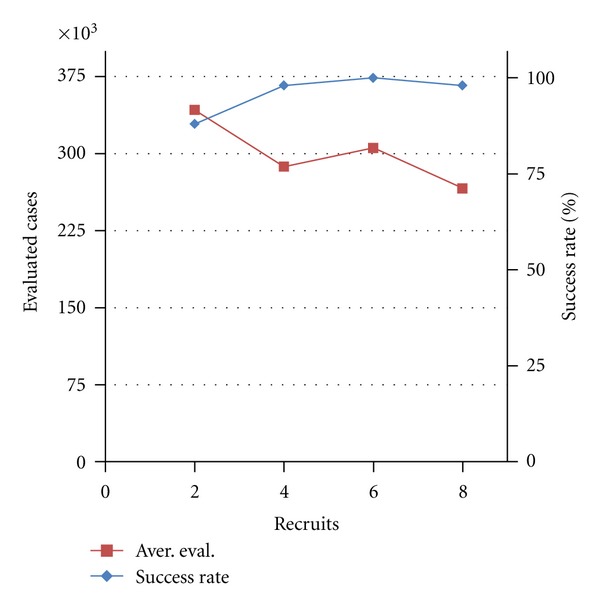
Static transposon tests. (a) Evolvability improves (and evolution speeds up) with number of genes in network. Other parameters are as follows: Popul = 8000; Reprod = 40%; Recrs = 2,4,6,8 (maximum number of recruits); Mut = 32%; Cross = 8%; EvalSum = 1000000.

The static transposon tests are generally slower to evolve than the static matrix case (Section 3.2), but faster than the dynamic matrix scenario (Section 3.3).  Figure 9 shows that static transposon tests do show the same trend of increasing evolvability with increasing network size.

#### 3.4.2. Dynamic Transposons

Here, we consider dynamic introduction of transposons, as illustrated in Figure 5. For simplicity, we initiated these computations with all members of the population invaded by transposons. Transposon dynamics are controlled by 2 parameters: TE growth,which controls the rate at which transposons can spread in a column of the *W* matrix (to a maximum length set by transposon length = 2 + (1 or 2); TE action, which controls how fast *W* ^*ab*^ elements decay given transposon infection.

**Figure 10:**
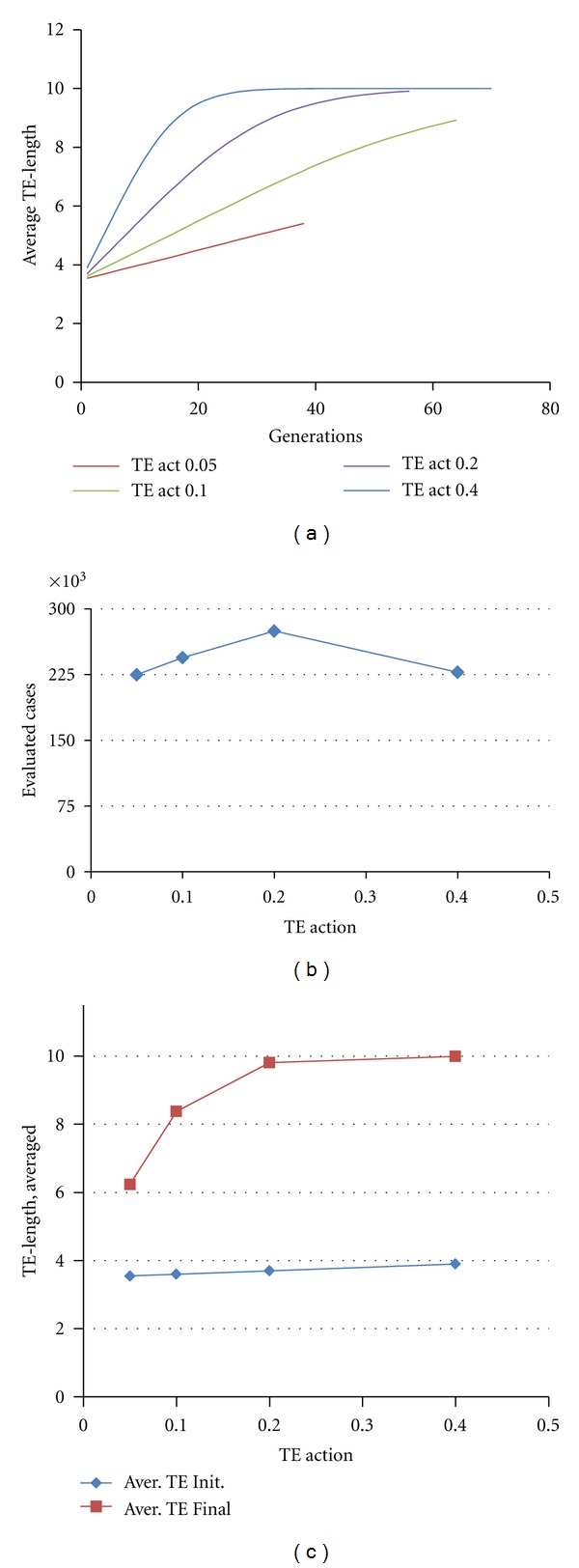
Dynamic transposons. (a) Examples of the growth of transposons (population average) during computational evolution, for different values of the transposon/transposition operators TE growth and TE action. (b) The dependence of evolvability on transposon activity. (c) Sustainable growth of transposon length during evolutionary search at different activity levels of the transposons. Other parameters are as follows: Popul = 8000; Reprod = 40%; Recrs = 8 (maximum number of recruits); Mut = 32%; Cross = 8%; EvalSum = 1000000; TE growth = TE action.

In general, we find that the efficacy of the evolutionary search in this scenario is comparable to the cases of fixed *W* matrix (Figure 7) or static transposons (Figure 9) (all simulations compared at GRN dimension of 10). Higher and lower TE action values may produce higher evolvability; midrange TE action may slow evolution (Figure 10(b)). As TE action is increased, the transposon length more quickly achieves the maximum length (Figures 10(a) and 10(c)). Similar results were seen for TE growth. As in Sections 3.2 and 3.3, the best scoring GRNs tend to coopt all available genes (make the largest possible GRN).

### 3.5. Spatial Patterns of Coopted Genes

**Figure 11:**
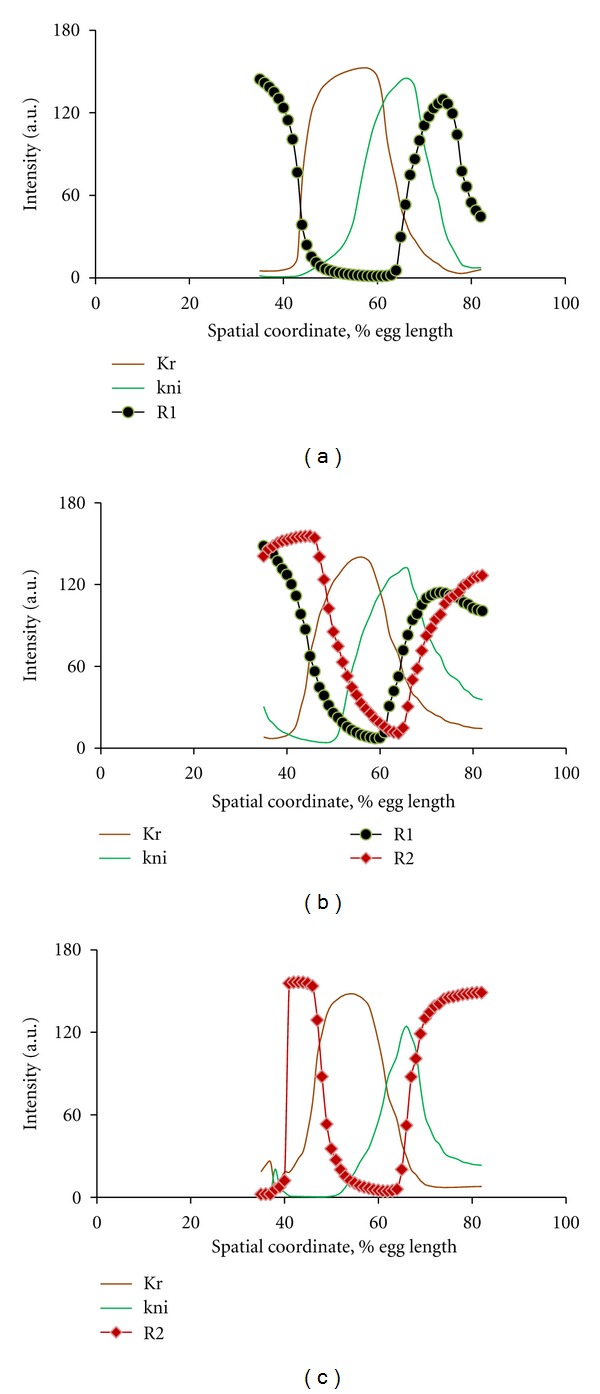
Representative examples of 2-gene models with recruits. (a) Recruit pattern is similar to gt or hb (black; c.f. Figure 3(a)). (b) Recruit patterns are similar to hb and gt (black and red; Cf. Figure 3(a)). (c) Recruit pattern looks like gt in reverse orientation (red; Cf. Figure 3(a)).

We found gene recruitment and functional incorporation into the GRNs to be quite general, regardless of the Gene Introduction mechanism. What sort of functionality do these recruited genes take in the network? We found recruited genes produced sophisticated spatial patterns with subdomains, influencing the spatial patterning of the obligatory two genes of the network (Kr & kni).  Figure 11 shows representative examples of such networks.

These patterns are reminiscent of the mature patterns of *Drosophila* gap genes and demonstrate how recruitment could supply new gap genes for an evolving segmentation network (as in the transition from short to long germ band mechanisms discussed in the Introduction).

Since the initial (Kr-kni) model networks lack the hb and gt regulators found in the real *Drosophila* 4-gene network, we were very curious whether the evolutionary computations might recapitulate hb- and gt-like patterns and functions. Indeed, the patterns of the coopted genes are usually reminiscent of anterior and posterior hb or gt domains (sometimes in reverse orientation). It could be that the evolutionary search is tending to fill in the missing gap patterns to generate the structure of the real, complete gap network (and better fit the real expression data). 

Our simulations show that outgrowth of 2-gene subnetworks via recruitment leads to cooption of genes which do recapitulate the patterns of real gap genes (i.e., gap genes which are part of the real segmentation network but are originally missing in the simulations). Our simulations, therefore, are an indication of how the gap gene network may have evolved to solve the particular problem of simultaneously forming properly positioned expression domains. In particular, our simulations may indicate how insect segmentation GRNs may have evolved from the primitive short germ mode to the derived long germ mode. 

### 3.6. Gene Networks under the Control of Two Gradients

We found that the GRN solutions described in Section 3.5 (in which the only external, nongap gradient was Bcd) were not robust to Bcd variability. This is in contrast with our observations from a similar computational evolution project [71], in which Bcd-robust solutions were found in a few percent of all good solutions (those that fit the expression data). The main differences are that the earlier project considered (i) *hb* and *Kr* as the pair of obligatory genes, and (ii) two maternal morphogenetic gradients as external inputs (Bcd and Cad). As noted above, some of the Bcd-only networks did recruit posterior-anterior gradients, perhaps to compensate a missing essential feature of the biological network. To determine the effect of the posterior gradient, we added Cad to the model (see Figure 3(b)). Computationally, this two-gradient version (Bcd-Cad) of the model behaved very similarly to the Bcd-only model. We will therefore focus here on the characteristics of the resultant GRNs. 

#### 3.6.1. Robustness of the Gene Networks with Two Gradients

**Figure 12:**
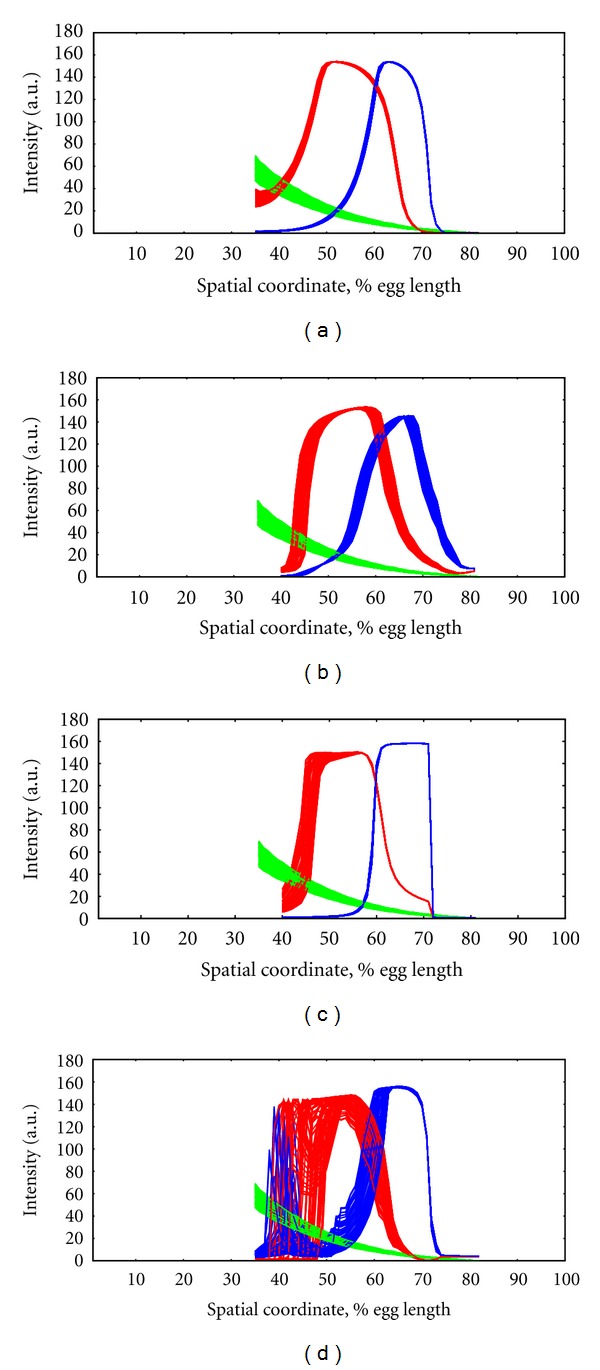
2-gradient model (Bcd-Cad) shows robustness to Bcd variability. Bcd—green; Kr—red; *kni*—blue. (a) Highly robust Kr and kni. (b) Nonrobust Kr and kni. (c) All borders are robust, except for anterior Kr. (d) Severe nonrobustness, especially for Kr, probably caused by bifurcations between GRN basins of attraction (multistability; c.f. [74]).

Addition of Cad to the network resulted in a number of the solutions displaying robustness to Bcd variability (Figure 12). Some of these showed higher robustness than is observed for real *Drosophila* segmentation genes (Figure 12(a); c.f. [33]). However, many good or even very good solutions (according to the fitness score for matching experimental expression patterns) can show no robustness to Bcd variability. These non-robust solutions can give Kr and kni variability as high as that for Bcd (Figure 12(b)); that is, Bcd variability is directly transmitted to its downstream targets, in contradiction to the observed severalfold reduction in variability seen in the data [94]. We see little correlation between goodness-of-fit to the expression data and robustness to Bcd variability; best-fit solutions can span from highly robust (Figure 12(a)), capable of filtering out Bcd variability nearly completely, to solutions unable to filter variability at all (Figure 12(b)).

It has been experimentally established that the position of each domain border of each gap gene pattern is under the control of different combinations of regulatory inputs from the other members of the segmentation network. The 2 obligatory genes (Kr and kni) in the model have two borders (anterior and posterior) each. Even for good-scoring solutions, there are cases where the *kni* border positions are robust but the Kr borders are less robust (even non-robust, Figure 12(d)). In many cases, the anterior Kr border is less robust than the posterior one (Figure 12(c)).

Our results indicate that robustness of the Kr-kni pattern depends on external gradients from both ends of the embryo, as provided by Bcd and Cad. We find that robustness can evolve relatively independently at each border. Hence, the positional error for each border can be relatively independent. This implies that whatever the mechanism of robustness for boundary precision, this may need to be evolved and established for each boundary, especially for systems such as *Drosophila* segmentation in which the combination of regulators controlling each boundary is unique.

## 4. Discussion

The main conclusion of this work is that GRN evolution tends to coopt all available genes. Network enlargement and functional redundancy of gene-gene connections do not prevent the cooption of new functional genes. With our Gene Introduction and Gene Withdrawal operators, we could directly investigate the effect of these rates on network outgrowth. If random mutation is also operating, Withdrawal rates can be significantly higher than Introduction rates before network outgrowth is halted. These findings are in agreement with the natural tendency towards gene recruitment found biologically (see Section 1).

Our modeling may offer insights into the evolution of insect segmentation. Our obligatory 2-gene network may have parallels to the short-germ mode of segmentation. The 2-gene model is not initially sufficient to fit the long-germ *Drosophila* data, but recruitment of additional genes can produce good fits to the long-germ mode. Introduction of a new gene often does not appear to directly increase the fitness. However, Withdrawal of the gene, after evolution to a good-scoring solution, can greatly reduce fitness, showing that it has acquired functionality in the network. Added genes do not generally provide structural redundancy, in which they “back up” a particular existing gene; rather, recruitment of a gene tends to alter the interactions in the original network. 

### 4.1. Redundancy and Robustness of Gene Networks

A notable feature of the early segmentation GRN is that it is under the control of not one but several maternally supplied gradients of transcription factors. For the core of the early GRN—the trunk gap genes—one should consider not only the primary morphogenetic gradient of Bcd, but also the maternal Hb and Cad gradients, and the terminal gradients (see review in [41]). We believe our evolutionary computations can shed some light on the functionality of this apparent redundancy of the biological gradients. In particular, we found that addition of the posterior Cad gradient was necessary in the present Kr-kni (obligatory) model to produce robustness to Bcd variability, in contrast to our earlier findings with a hb-Kr model [71]. This indicates that while particular 2-gene subnetworks may have evolved with robustness to Bcd variability (in agreement with recent theoretical work, [102]), other 2-gene pairs may require additional gradient input to form patterns that are robust to variability. Our present results begin to characterize these variations in robustness between gene pairs, and the role multiple gradients may play in creating robustness in the complete *Drosophila* long-germ segmentation network. 

With the Bcd-Cad model, robustness to Bcd variability can take a variety of forms, from all Kr and kni borders being very precise to cases in which particular borders show much different robustness than others. We feel this reflects the biological nature of the problem, in which different borders are under different regulatory factors. By comparing the present results to our prior work on the hb-Kr module, and extending our approach to investigate other gap pairs and robustness to variability in other gradients, such as Cad and maternal Hb, our modeling can offer insight into the ways in which these factors interact to confer local spatial precision, and insight into how these interactions evolved. For example, solutions which fit the data and are robust (in this and our prior work) tend to be found much less frequently than solutions which simply fit the data. Evolutionarily, solutions, for example to long-germ segmentation, may have evolved readily, but search for solutions with robustness to variability may take much longer. This frequency of robust solutions will be explored more fully in future work.

### 4.2. Forced Evolution by Artificial Transposons

The method of forced GRN evolution by artificial transposons is described in further detail in [83]. Together with the present work, we are gaining insights into some of the diverse features of the coevolution of GRNs and their transposons. 

**Figure 13:**
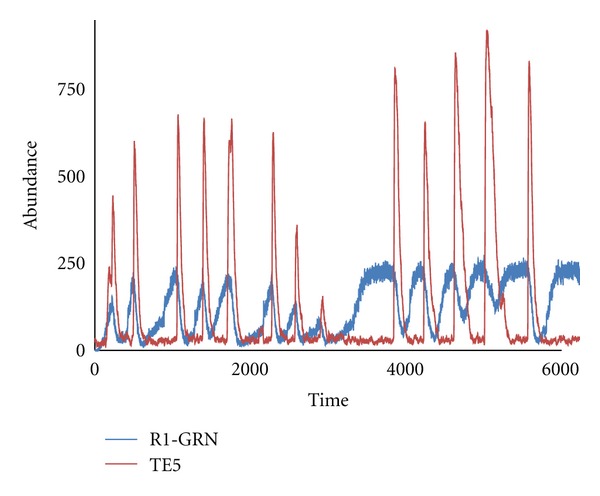
Host-transposon coevolution. Oscillatory dynamics of the different “strains” of GRNs and transposons under the restriction of possible gene recruits to 1 (R1). The initial 4-gene network (GRNini = GRN_4_) abundance oscillates with the one-recruit network (4 + 1 genes, or R1-GRN). The oscillations are accompanied by short bursts of TE_5_ transposon abundance.

For example, preliminary computations making the 4 core gap genes (*gt*, *hb*, *Kr*, and *kni*) obligatory and limiting the number of potentially recruited genes to 1 (R1) show parallels between GRN-transposon coevolution and host-parasite (or predator-prey) dynamics.  Figure 13 shows a time course of these dynamics. Given an initial population of GRNs (GRN_ini_ = GRN_4_), a primary invasion of the initial transposons (TE_ini_ = TE_4_, i.e., transposons of length = 4) spreads through the initial population. TE_4_ infection gradually reduces the GRN_4_ fitness score. Transposons in this case are defined as growing and transmitting. As a result of the selection pressure on GRN_4_, recruitment allows R1 networks (which cannot be infected by TE_4_) to become prevalent in the population. However, due to transposon growth, transposons of length 5 (TE_5_) soon appear and begin to infect R1-GRNs. The infection gradually decreases the R1-GRN scores while increasing the prevalence of TE_5_ (Figure 13, early times). The decreasing proportion of TE_4_ in the population makes GRN_4_ relatively fit again, and the TE_5_-infected R1-GRNs begin to be eliminated by selection and replaced by GRN_4_s (which are defined in the model to be steadily supplied from an “external reservoir”). In this way, the population becomes rejuvenated and free of TE_5_. The prevalence of GRN_4_, however, makes the population susceptible to TE_4_ infection again; the cycle repeats, and we observe oscillations in the abundance of the GRN and TE species (Figure 13). Such coevolutionary oscillations are wellknown from host-parasite or predator-prey dynamics. Of interest for future work is the nature of the irregularity in the oscillations, for example, understanding why R1-GRN and TE_5_ start in-phase and gradually settle into an out-of-phase relation (Figure 13; e.g., do the initial dynamics point to a pool of evolved TE_5_ “waiting” for the host R1-GRN to evolve, with subsequent dynamics more tightly codependent?). 

We believe that these scenarios of GRN-transposon coevolution could be used as a new tool in forced GRN computational evolution. Specifically, it is a promising mechanism for gentle and indirect forced GRN evolution. The observed oscillations could be useful in overcoming the very general problem of premature convergence in evolutionary searches. 

### 4.3. Discrete versus Continuous Approaches (Boolean versus ODE/PDE Models)

A great deal of the work on evolutionary computations of GRNs has been done with discrete-value Boolean approaches, in which genes are either “on” or “off.” While these approaches can be fast and lead to general conclusions on evolutionary dynamics [60], they can be insufficient for addressing real biochemical networks, where use of continuous differential equations may be more appropriate. For GRNs reverse-engineered to experimental data, evidence suggests that continuous models are more faithful to known interactions than Boolean models. For example, for AP segmentation in *Drosophila*, Perkins et al. [103] compared two discrete logical models with two continuous reaction-diffusion (RD) models and found both RD models fit the data better than the logical models. 

Another caution is that the evolutionary landscape of GRNs can be quite different depending on whether a discrete or continuous approach is used. Using a discrete approach, Ciliberti with coauthors [60] suggested that the collection of GRNs which create a particular phenotype (e.g., expression pattern) form a neutral basin in the fitness landscape, such that drift within the basin allows for a neutral means of sampling different phenotypic variations (at the “borders” of the basin). However, this discrete approach does not address the natural continuous variation of gene-gene interaction parameters (due, e.g., to tuning of enzymatic co-factors or complex coregulation by multiple transcription factors). Our evolutionary searches indicate that very small differences in these parameters can produce very different phenotypes (e.g., robust versus non-robust to maternal variability). Our results suggest that the achievement of robust GRNs in a continuous evolutionary search can be quite rare, and that such solutions can be quite isolated, reflecting a complex fitness landscape which is far from neutral. Continuous descriptions are needed to capture the size and complexity of the genotype space. Such complexity is also indicated by theoretical studies of continuous-GRN parameter spaces showing multistability (e.g., [104]). 

In addition to a more complex description of the evolutionary landscape, modeling at the PDE level in this work has allowed us to specifically investigate the continuous variation of the Bcd gradient, for testing robustness of the GRNs to maternal variability; as well as allowing us to model the effect of transposons as a gradual zeroing of network interactions, rather than as discrete knockouts. The qualitative differences between the discrete and continuous approaches, and the different questions that can be asked with each, warrant careful consideration when developing models or analyzing results. 

## Figures and Tables

**Table 1:** Elements of *W* ^*a*0^ kept constant in the static transposon tests.

Matrix dimension	*W* ^10^	*W* ^20^	*W* ^30^	*W* ^40^	*W* ^50^	*W* ^60^	*W* ^70^	*W* ^80^	*W* ^90^	*W* ^90^
4	0	33	67	100						
6	0	20	40	60	80	100				
8	0	14	29	43	57	71	86	100		
10	0	11	22	33	44	56	67	78	89	100
